# Humanized bone facilitates prostate cancer metastasis and recapitulates therapeutic effects of zoledronic acid in vivo

**DOI:** 10.1038/s41413-019-0072-9

**Published:** 2019-10-21

**Authors:** Marietta Landgraf, Christoph A. Lahr, Alvaro Sanchez-Herrero, Christoph Meinert, Ali Shokoohmand, Pamela M. Pollock, Dietmar W. Hutmacher, Abbas Shafiee, Jacqui A. McGovern

**Affiliations:** 1 Centre in Regenerative Medicine, Institute of Health and Biomedical Innovation, Queensland University of Technology, Brisbane, Australia; 2 School of Biomedical Science, Institute of Health and Biomedical Innovation, Translational Research Institute, Queensland University of Technology, Brisbane, Australia; 3 Australian Research Council (ARC) Training Centre in Additive Biomanufacturing, Queensland University of Technology, Brisbane, Australia; 4 UQ Diamantina Institute, Translational Research Institute, The University of Queensland, Brisbane, QLD Australia

**Keywords:** Bone cancer, Bone, Bone cancer

## Abstract

Advanced prostate cancer (PCa) is known for its high prevalence to metastasize to bone, at which point it is considered incurable. Despite significant effort, there is no animal model capable of recapitulating the complexity of PCa bone metastasis. The humanized mouse model for PCa bone metastasis used in this study aims to provide a platform for the assessment of new drugs by recapitulating the human–human cell interactions relevant for disease development and progression. The humanized tissue-engineered bone construct (hTEBC) was created within NOD-scid IL2rg^null^ (NSG) mice and was used for the study of experimental PC3-Luc bone metastases. It was confirmed that PC3-Luc cells preferentially grew in the hTEBC compared with murine bone. The translational potential of the humanized mouse model for PCa bone metastasis was evaluated with two clinically approved osteoprotective therapies, the non-species-specific bisphosphonate zoledronic acid (ZA) or the human-specific antibody Denosumab, both targeting Receptor Activator of Nuclear Factor Kappa-Β Ligand. ZA, but not Denosumab, significantly decreased metastases in hTEBCs, but not murine femora. These results highlight the importance of humanized models for the preclinical research on PCa bone metastasis and indicate the potential of the bioengineered mouse model to closely mimic the metastatic cascade of PCa cells to human bone. Eventually, it will enable the development of new effective antimetastatic treatments.

## Introduction

Prostate cancer (PCa) is the second most frequently diagnosed cancer among the male population in industrialized countries.^1^ Approximately 50% of patients suffering from advanced PCa experience at least one complication within 2 years due to the development of bone metastases.^2^ These so-called skeletal-related events (SREs) are associated with poor prognosis, severe bone pain, pathological fractures, and imply in most cases the incurability of the disease.^3,4^ In fact, it is rather the health implications of bone metastases developing in later disease stages than the primary tumor itself that accounts for the mostly lethal outcome of PCa.^3,5^ The current gold standard of therapy for patients with PCa metastases is a combination of Taxane and corticosteroids, e.g., Docetaxel and Prednisone.^6^ The 5-year relative survival rate for metastatic PCa patients is only 28%, while it is 100% for patients with localized disease.^7^

Shiozawa et al.^8^ and others demonstrated that, amongst others, molecular changes in the bone microenvironment can lead to enhanced cancer cell proliferation and tumor recurrence or therapy resistance.^9^ Consequently, emerging therapeutic approaches, like bisphosphonates^10^ or the human-specific monoclonal antibody (Ab) Denosumab,^11^ were developed not only to target cancer cells but also the bone microenvironment to fight PCa bone metastases.^12^ Both agents target the Receptor Activator of Nuclear Factor Kappa-Β Ligand (RANK-RANKL) system and have already shown advantageous effects on the reduction of breast and prostate cancer bone metastases in clinical trials.^2,13^ Bisphosphonates, in general, have a high affinity for bone tissue and inhibit bone resorption caused by osteoclasts.^14^ Zoledronic acid (ZA) is a highly potent nitrogen-containing bisphosphonate and was clinically approved for the treatment of bone metastases from solid tumors in 2002.^15^ Various direct and indirect antitumor effects such as inhibition of angiogenesis, tumor cell proliferation, adhesion, and invasion have been reported for ZA.^16^ It was also shown that ZA can act in synergy with cytotoxic drugs^17^ and enhances tumor cell apoptosis and immune surveillance.^18^ In addition, ZA is applied as a once-yearly injection (5 mg) for prevention and treatment of osteoporosis.^19^ In contrast, Denosumab binds and neutralizes RANKL, thereby reducing bone resorption and inhibiting osteoclast recruitment and maturation.^20,21^ In 2011, Denosumab was approved by the European Medicines Agency and the United States Food and Drug Administration as osteoprotective therapy for patients at high risk of fracture due to bone metastases from solid tumors like PCa.^22^ Denosumab binds circulating RANKL in the extracellular space instead of bone tissue, which allows for rapid onset, fast and fully reversible offset of the therapy.^20^ Due to this more precise mode of action compared with bisphosphonates, Denosumab is used in patients where other available treatments have failed or are not feasible.^20^ In a randomized double-blind study in men with castration-resistant PCa, Denosumab (120 mg every 4 weeks, subcutaneous) performed better in the prevention and treatment of SREs than ZA (4 mg every 4 weeks, intravenous).^23^

The natural heterogeneity of PCa makes the choice of the treatment strategy very difficult.^24^ To gain more knowledge about the molecular factors triggering and influencing PCa bone metastasis and to develop innovative treatment strategies, various in vivo models were established.^25^ As Denosumab binds exclusively to human RANKL and not murine RANKL, the development of a genetically engineered mouse model (huRANKL mice) for the preclinical in vivo evaluation of this Ab was essential.^26^ However, even though only one molecule was modified in this mouse model, the genetic alteration had a considerable impact on the whole physiology of the animals. HuRANKL mice were up to 20% smaller than wild-type mice at 4 months of age, had significantly lower Tartrate-Resistant Acid Phosphatase 5b (TRACP-5b) serum levels and an altered bone phenotype with reduced osteoclast and osteoblast surfaces.^26,27^ These limitations of the model influence the general comparability of the results with other mouse models as well as the clinical translatability. In our group, the humanized tissue-engineered bone construct (hTEBC) mouse model was established and used as a platform to gain insights into the development of primary bone cancer as well as prostate and breast cancer bone metastases.^28–30^ The bioengineering approach allows us to provide a humanized bone niche without the need for genetic modifications, which may potentially have a large impact on mouse physiology. In addition, it enables us to recapitulate the effect of human-specific therapeutics in a humanized tissue microenvironment, which comprises multiple human cues relevant to disease development and progression, not only one specific factor. Eventually, this enables a clearer understanding of human PCa cell interactions with the bone microenvironment and to more reliably and comparable evaluate new drug candidates against PCa bone metastases.^31^

In the current study, we employed our platform to investigate the impact of the clinically approved treatment regimens of ZA and Denosumab on PCa bone metastasis. We were able to show that human PC3 cells stably expressing luciferase (PC3-Luc) show superior growth in the hTEBC compared with the mouse femur. Furthermore, our model is capable of recapitulating the therapeutic effects of ZA, but not of Denosumab.

## Results

### The therapeutic effects of ZA and Denosumab in a humanized mouse model for bone metastasis research

**Fig. 1:**
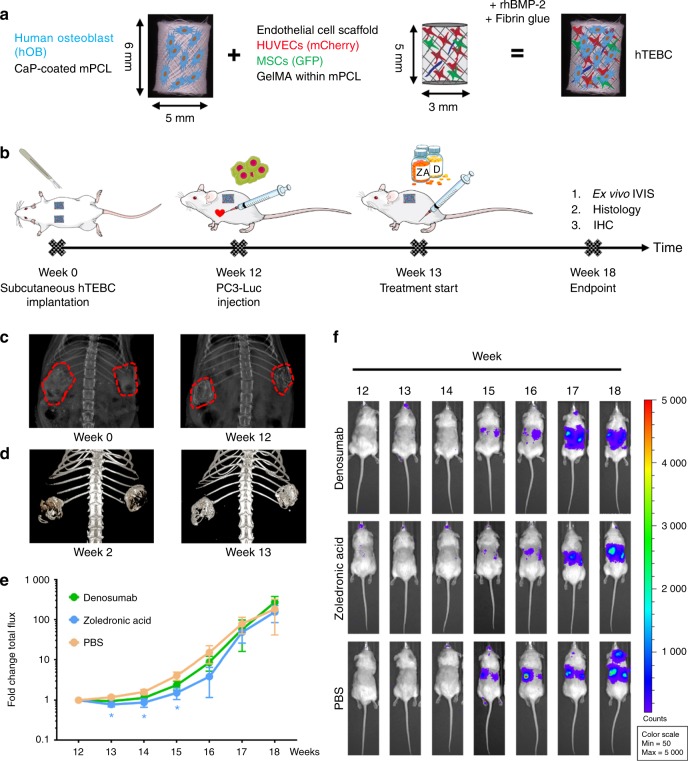
Bone formation at the humanized tissue-engineered bone construct (hTEBC) and effect of zoledronic acid (ZA) and Denosumab on the outgrowth of experimental PC3-Luc metastases in vivo. **a** Schematic manufacturing process of the hTEBC. **b** Timeline of the PC3-Luc in vivo study. **c**, **d** Representative images of in vivo bone formation, monitored by x-ray (**c**) and in vivo CT (**d**). Imaging throughout the duration of the study showed an increase of mineralized tissue at the hTEBC implantation site. **e** In vivo BLI total flux fold changes over time. ZA reduced the metastatic load significantly between week 13 and 15 when compared with PBS. Animals treated with Denosumab also showed reduced PC3-Luc tumor burden between week 13 and 17, however without significance. From week 17 to 18, no significant difference between the three experimental groups was apparent. Each data point represents mean ± SEM (*n* = 6 for Denosumab; *n* = 5 for ZA and PBS). *P*-values were calculated using a UNIANOVA model (*P ≤ 0.05). **f** Representative in vivo BLI time course for each treatment group

To provide a humanized bone microenvironment for human PC3-Luc cells, we bioengineered hTEBCs.^32^ The hTEBC consisted of a tubular medical-grade polycaprolactone (mPCL) scaffold seeded with human osteoblasts (hOBs) and a pre-vascularized gelatin methacryloyl (GelMA)-core containing human umbilical vein endothelial cells (HUVECs) and multipotent mesenchymal stromal cells (MSCs) (Fig. 1a). Together with fibrin glue and recombinant human bone morphogenic protein 2 (rhBMP-2), the hOB scaffold and the GelMA core formed the hTEBC, which was subcutaneously implanted into male NOD-scid IL2rg^null^ (NSG) mice (Fig. 1b). Ultrasound-assisted intra-cardiac injection of PC3-Luc cells into the left ventricle was performed after 12 weeks of in vivo bone formation and led to the development of experimental PCa metastases. The PCa metastases were subsequently treated twice per week with ZA or Denosumab for six consecutive weeks. Throughout the study bone formation was monitored by x-ray (Fig. 1c) and in vivo computed tomography (CT) (Fig. 1d) and showed an increase of mineralized tissue at the hTEBC implantation site. Distribution and outgrowth of human PC3-Luc metastases were studied using in vivo bioluminescence imaging (BLI). Fold changes of the average total flux over time revealed a significant therapeutic effect of ZA in week 13 and 14 by reducing the initial tumor burden to 0.78-fold (*P* = 0.015) and 0.87-fold (*P* = 0.034), respectively (Fig. 1e). Denosumab appeared to decrease the tumor burden in week 13 to 0.95, but without a statistically significant difference when compared with PBS (1.19-fold). In week 15, ZA treatment led to a significantly lower metastatic burden (1.52-fold, *P* = 0.016) compared with Denosumab (2.38-fold) and PBS (4.09-fold). This pattern was still apparent in week 16 where animals treated with ZA showed the least increase in total flux values (3.87-fold), followed by Denosumab (8.70-fold) and PBS (15.17-fold), although this was not statistically significant. At week 17 and 18, neither ZA (48.29- and 154.22-fold) nor Denosumab (57.04- and 271.57-fold) had an observable therapeutic effect when compared with PBS (78.78- and 182.11-fold). Representative BLI time courses of the study are shown next to the graph (Fig. 1f). Overall, these results indicate that ZA exhibits a more prominent antimetastatic effect on PC3-Luc cells than Denosumab in our in vivo model.

### ZA reduces the metastatic load in the murine liver

**Fig. 2:**
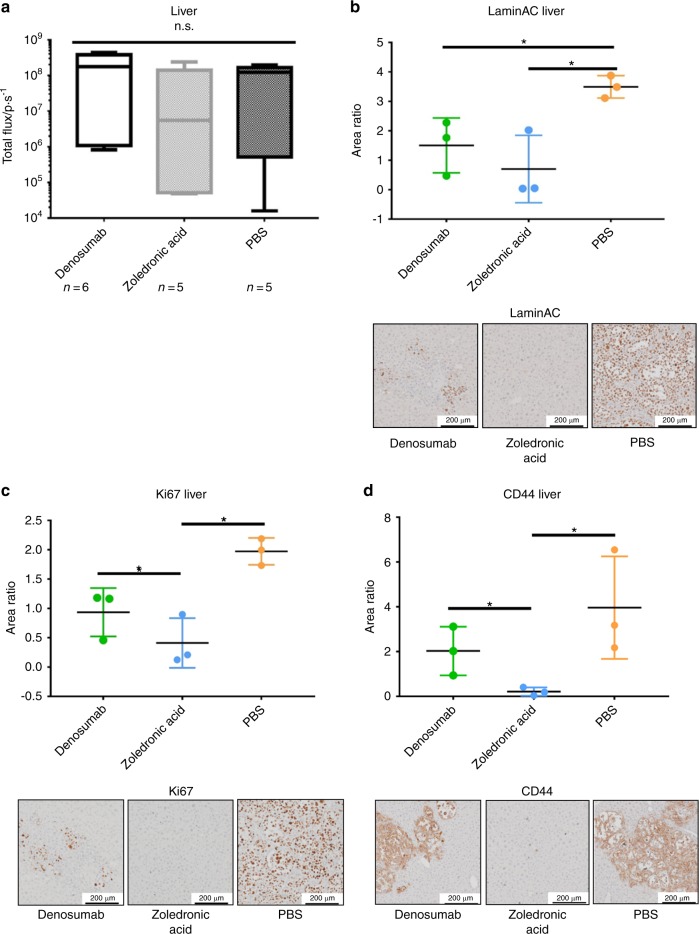
Zoledronic acid (ZA) reduces the metastatic burden in the liver. **a** Ex vivo bioluminescence imaging (BLI) of livers shows a reduced metastatic load after ZA treatment compared with Denosumab and PBS, however without statistical significance. *P*-values were calculated using a UNIANOVA model (*n* = 6 for Denosumab; *n* = 5 for ZA and PBS). **b** Immunohistochemistry (IHC) staining of human-specific LaminAC revealed the presence of significantly less human PC3-Luc cells in liver tissue of animals treated with Denosumab and ZA compared with the PBS control group. *P*-values were calculated using a UNIANOVA model (*P ≤ 0.05; *n* = 3 for each group). Representative images of human-specific LaminAC IHC on murine livers are below the graph. **c** IHC staining of Ki67, a marker that is commonly used for the detection of proliferative cancer cells,^64^ confirmed a significant therapeutic effect of ZA on PC3-Luc liver metastases in comparison to Denosumab and PBS. *P*-values were calculated using a UNIANOVA model (*P ≤ 0.05; *n* = 3 for each group). Representative images of Ki67 IHC on murine livers are below the graph. **d** IHC staining of human-specific CD44 showed a significantly lower amount of human PC3-Luc cells in murine liver tissue after ZA treatment compared with the Denosumab and PBS group. *P*-values were calculated using a UNIANOVA model (*P ≤ 0.05; *n* = 3 for each group). Representative images of human-specific LaminAC IHC on murine livers are below the graph

As in vivo BLI did not reveal significant differences between the two experimental groups and the control group at later time points, ex vivo BLI and immunohistochemistry (IHC) were chosen to visualize any differences between treatments. Therefore, comparison of ex vivo BLI results with IHC staining for human-specific LaminAC, Ki67, and human-specific CD44 of mouse livers as independent organs of the research question on bone metastasis was conducted (Fig. 2). Ex vivo BLI analysis (*n* = 6 for Denosumab; *n* = 5 for ZA and PBS) did not show a significant difference in liver metastasis between the three groups (Fig. 2a). However, staining for human-specific LaminAC indicated a significantly higher tumor burden in the liver tissue of animals treated with PBS compared with ZA (*P* = 0.038) and Denosumab (*P* = 0.042) (Fig. 2b). Furthermore, the proliferation marker Ki67 showed a significant reduction of actively proliferating PC3-Luc cells after ZA treatment compared with Denosumab (*P* = 0.034) and PBS (*P* = 0.023) (Fig. 2c). Those results were confirmed by staining for human CD44 where significantly less PCa-Luc cells were detected in the ZA group compared with PBS (*P* = 0.020) and Denosumab (*P* = 0.040) (Fig. 2d). These findings suggest that complementary histology and IHC provide more accurate results than BLI alone when dissecting effects on subjacent tissues like bone metastases, which are covered by dense bone and a considerable layer of connective tissue.

### ZA reduces metastatic load and increases bone formation in hTEBCs

**Fig. 3:**
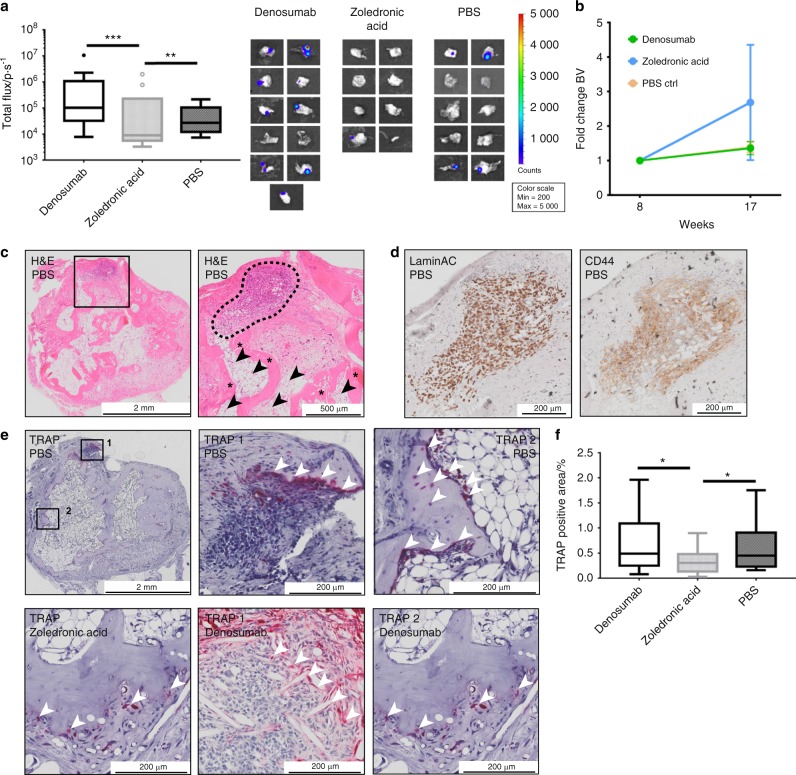
Zoledronic acid (ZA), but not Denosumab, reduces metastatic load and increases bone formation in humanized tissue-engineered bone constructs (hTEBCs). **a** Ex vivo bioluminescence imaging (BLI) revealed a significant reduction of the metastatic load in hTEBCs by ZA compared with Denosumab and PBS. The Tukey method was used to determine outliers (shown as individual data points). One data point for Denosumab and two for ZA were excluded for calculation of *P*-values using a UNIANOVA model (**P ≤ 0.01, ***P ≤ 0.001; *n* = 11 for Denosumab; *n* = 8 for ZA; *n* = 10 for PBS). BLI images of included hTEBCs are shown to the right of the graph. **b** Fold change of the bone volume (BV) at the hTEBC between week 8 (before cancer cell injection) and 17. hTEBCs in mice treated with Denosumab or PBS maintained a constant BV after cancer cell injection. Only animals treated with ZA showed an increase in BV after PC3-Luc injection of ~2.5-fold, however without statistical significance. **c** The histology (H&E) overview section (left panel) confirmed a PC3-Luc metastatic lesion within the hTEBC (black square). In the zoom in (right panel) the bone marrow compartment (black arrowheads) and mineralized tissue (asterisks) resembling a human bone niche with trabecular and cortical bone are apparent. **d** Immunohistochemistry (IHC) staining for human LaminAC confirmed the human origin of metastases in hTEBCs (left panel). IHC staining for CD44 confirmed that the lesion originated from prostate cancer cells (right panel). **e** Tartrate-resistant acid phosphatase (TRAP) staining of the hTEBC revealed an invasive line of TRAP-positive cells of the osteolytic PC3-Luc lesion in the PBS and the Denosumab group (TRAP1 respectively, white arrow heads). In addition, physiological bone turnover was observed at the interface of the human mineralized tissue within the hTEBC for all three experimental groups (TRAP2 for PBS and Denosumab group, white arrow heads). **f** Quantification of the TRAP-positive tissue area for each treatment group showed a significant reduction of osteoclast activity after treatment with ZA, but not for the Denosumab and PBS group. *P*-values were calculated using a UNIANOVA model (*P ≤ 0.05; *n* = 15 for Denosumab; *n* = 16 for ZA; *n* = 13 for PBS)

To elucidate the metastatic burden of PC3-Luc cells in the hTEBCs in more depth, ex vivo BLI was performed after explantation (Fig. 3a). The hTEBCs of animals treated with ZA had a mean total flux of 1.27 × 10^4^ ± 4.99 × 10^3^ p·s^−1^ (mean ± SEM, *n* = 8), which was significantly lower than both Denosumab (4.04 × 10^5^ ± 2.19 × 10^5^ p·s^−1^; mean ± SEM; *n* = 11; *P* = 0.000 5) and PBS (6.38 × 10^4^ ± 2.24 × 10^4^ p·s^−1^, mean ± SEM, *n* = 10, *P* = 0.01). Values from three hTEBCs (one of the Denosumab and two of the ZA group) were excluded from the statistical analysis as they were considered as outliers according to the Tukey method.^33^ The BLI images of hTEBCs included in the analysis are shown to the right of the graph. When comparing the fold change in BV shortly before (week 8) and 5 weeks after PC3-Luc injection, it was apparent that in animals treated with Denosumab (1.36-fold) or PBS (1.39-fold) the BV was maintained with no significant difference between the groups (Fig. 3b). In contrast, in the ZA treatment group the BV increased 2.68-fold. Histology (H&E) of hTEBCs confirmed the presence of metastatic PC3-Luc lesions (black square in the left panel, dashed black line in the right panel; Fig. 3c). At higher magnification, it was apparent that the PC3-Luc cells were growing in a hematopoietic microenvironment comprised of bone marrow (black arrowheads) and mineralized tissue (asterisks) resembling a physiological bone niche within the hTEBC. IHC staining of the lesion with human-specific LaminAC (left panel) confirmed the human origin of the lesions and staining for CD44 (right panel) identified it as PCa metastasis derived from PC3-Luc cells (Fig. 3d). Tartrate-resistant acid phosphatase (TRAP) staining was used to detect osteolytic activity within the hTEBC (Fig. 3e). An osteolytic line at the invasive front of the PC3-Luc lesions in the hTEBC of representative animals from the PBS and Denosumab group (TRAP 1, mid panel) was identified. In addition, areas with normal bone turnover along the interface of the mineralized tissue within the hTEBC were observed for all three groups (ZA left panel, PBS and Denosumab TRAP 2, right panel). This confirms the generation of a physiologically relevant bone organ as well as a homing site for metastatic PCa cells. Quantification of the TRAP staining associated with metastasis only showed a significant effect on osteoclast activity of ZA with only 0.34% ± 0.06% (mean ± SEM, *n* = 16, *P* = 0.043 to Denosumab and PBS, respectively) TRAP-positive area (Fig. 3f). In contrast, hTEBCs of animals treated with Denosumab reached 0.74% ± 0.16% (mean ± SEM, n = 15) TRAP-positive tissue area and of mice receiving PBS 0.63% ± 0.14% (mean ± SEM, *n* = 13). These results together indicate that ZA is exhibiting a significant inhibitory effect on osteoclasts, reducing PC3-Luc metastatic load in the hTEBCs.

### Neither ZA nor Denosumab shows an effect on the PC3-Luc tumor burden in the murine femur

**Fig. 4:**
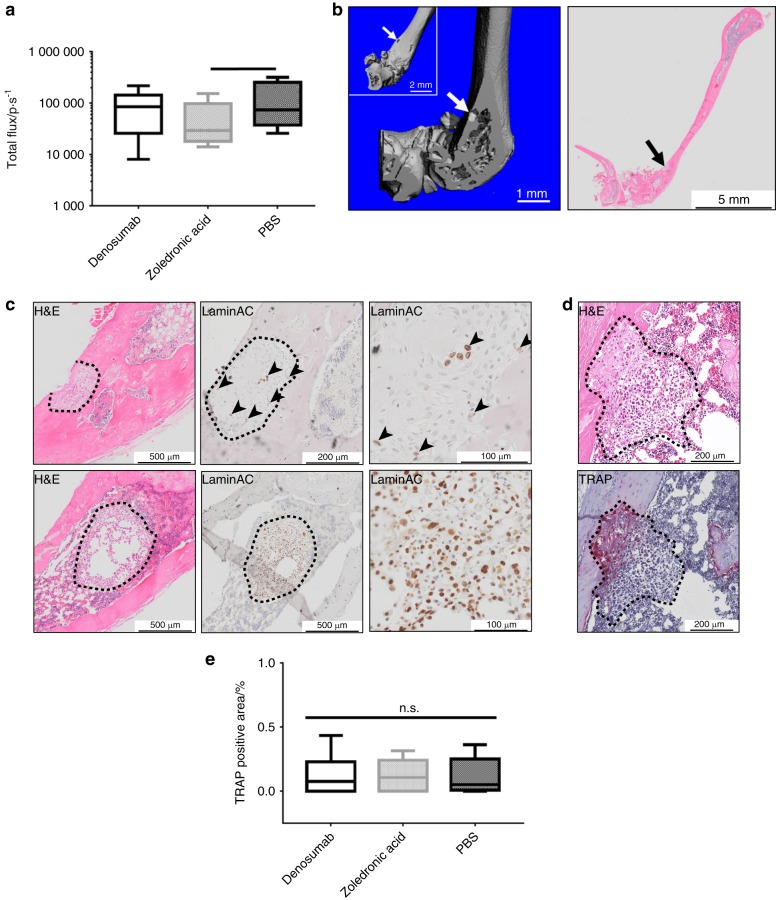
Neither zoledronic acid (ZA) nor Denosumab shows an effect on the PC3-Luc tumor burden in the mouse skeleton represented by the femur. **a** Ex vivo bioluminescence imaging (BLI) indicated a significant reduction of the metastatic load in the mouse femur by ZA when compared with PBS. Femora of animals treated with Denosumab showed a similar signal intensity as the PBS group, however without a significant difference to the ZA group. *P*-values were calculated using a UNIANOVA model (*P ≤ 0.05; *n* = 6 for Denosumab; *n* = 5 for ZA and PBS). **b** Representative image of a PC3-Luc bone lesion in the left femur of a mouse of the PBS control group confirmed by ex vivo µCT (left panel) as well as histology (H&E, right panel). The lesion penetrates the cortical shell of the mouse femur, exposing the trabecular bone compartment to the surrounding tissue. **c** Histology (H&E) and immunohistochemistry (IHC) for human-specific LaminAC revealed and confirmed the presence of metastatic lesions in the murine bone (upper panel, representative image from the PBS group) as well as in the bone marrow (lower panel, representative image from the Denosumab group) of the mouse femur. In the bone marrow lesion only very few cells of the entire metastatic area still expressed the human-specific marker LaminAC. **d** Histology (H&E) and tartrate-resistant acid phosphatase (TRAP) staining confirmed a TRAP-positive invasive line for metastases growing from the bone marrow into the cortical bone of the mouse femur (representative image from the Denosumab group). **e** Quantification of the TRAP-positive area for each treatment group indicated no significant difference between the effects of ZA, Denosumab or PBS treatment on the osteoclast activity in the mouse femur. *P*-values were calculated using a UNIANOVA model (*P ≤ 0.05; *n* = 3 for each group)

Ex vivo BLI of the murine left leg (femur and tibia) as representative for the mouse skeleton^34^ showed only a significant metastasis-reducing the effect of ZA (5.25 × 10^4^ ± 2.58 × 10^4^ p·s^−1^, mean ± SEM, *n* = 5, *P* = 0.039) when compared with PBS (1.31 × 10^5^ ± 5.43 × 10^4^ p·s^−1^, mean ± SEM, *n* = 5; Fig. 4a). Denosumab (9.09 × 10^4^ ± 3.08 × 10^4^ p·s^−1^, mean ± SEM, *n* = 6) reduced the metastatic load slightly as well, but without statistical significance. Osteolytic lesions in the murine femur were confirmed with ex vivo µCT (left panel, representative images of the PBS group, Fig. 4b). PC3-Luc metastases were mainly located at the distal metaphysis of the femur, growing from the bone marrow into the bone of the femur and eventually protruding the cortical bone. The metastatic lesions were confirmed by histology (H&E) of longitudinal sections of the murine femur (right panel). Further characterization by histology (H&E) and IHC staining of LaminAC confirmed the presence of bone (upper row, representative image of the PBS group) as well as bone marrow metastases (lower row, representative image of the Denosumab group) in the murine femur (Fig. 4c). The proportion of LaminAC positive cells (black arrowheads) was smaller in the bone metastases compared with the bone marrow metastases. Metastatic lesions invading the cortical bone from the murine bone marrow compartment were confirmed by histology (H&E, upper panel, representative image of the Denosumab group, Fig. 4d). TRAP staining showed an osteolytic line at the invasive front of the PC3-Luc lesion (lower panel, representative image of the Denosumab group) similar to the observations in the hTEBC (Fig. 4e). Quantification of the TRAP-positive area associated with metastatic PC3-Luc lesions and not physiological bone turnover in the murine femora revealed considerably lower values compared with the hTEBCs (ZA 0.34% ± 0.06%, Denosumab 0.74% ± 0.16%, PBS 0.63% ± 0.14%; mean ± SEM; Fig. 4f) and no significant effect between the three groups (ZA 0.11% ± 0.04%, Denosumab 0.13% ± 0.06%, PBS 0.11% ± 0.05%; mean ± SEM, *n* = 3 for all three groups; Fig. 4e). These findings point toward a different behavior of PC3-Luc cells in the murine compared with the humanized bone microenvironment regarding general cell biology and marker expression as well as the therapeutic response to the applied treatments.

### PC3-Luc cells preferentially grow in hTEBCs

**Fig. 5:**
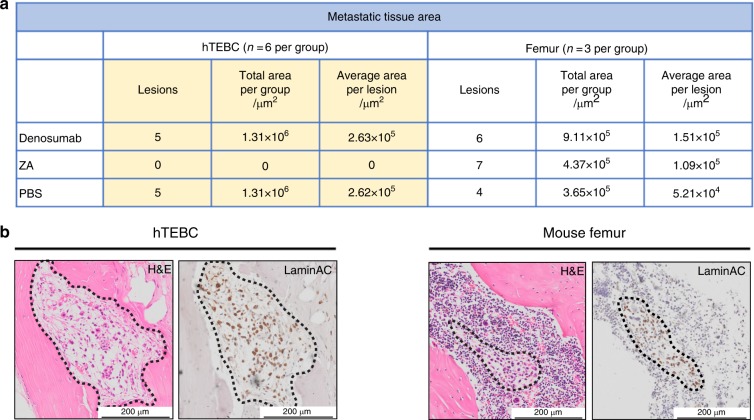
PC3-Luc cells preferentially grow in humanized tissue-engineered bone constructs (hTEBCs). **a** The number of metastatic lesions, total metastatic tissue area and average size per lesion was determined for hTEBC (*n* = 6 per group) and murine femur (*n* = 3 per group) using H&E, LaminAC, and TRAP stained tissue sections, which were analysed with the image analysis software Visiopharm. ZA diminished PC3-Luc metastases in the hTEBC, but not in the mouse bone. Denosumab was not capable of reducing the number of metastases in the hTEBC nor in the murine femur. Overall, the total metastatic area, as well as the average size per metastatic lesion, was bigger for the hTEBC than for the mouse femur. **b** Representative histology (H&E) and LaminAC immunohistochemistry (IHC) images of PC3-Luc cancer cell lesions in hTEBC (left panel) and mouse femur (right panel) from the PBS control group, indicating the increased size of metastases found in hTEBCs compared with the murine bone

Semi-automated image analysis of hTEBC and femur tissue sections further confirmed the difference of PC3-Luc cell characteristics induced through the humanized and the murine bone microenvironment (Fig. 5a). PC3-Luc lesions were identified by aberrant tissue and cell morphology, such as enlarged nuclei and irregular cell size and shape^35^ on H&E sections and confirmed through positive staining for human-specific LaminAC or TRAP. The metastatic area was determined using the histopathology image analysis software Visiopharm. ZA was able to diminish PC3-Luc lesions in hTEBCs (0 lesions, *n* = 6), but not in the murine femur (7 lesions, *n* = 3), indicating altered behavior and susceptibility of this PCa cell line in the host bone microenvironment. Denosumab and PBS did not have an effect on the establishment of PC3-Luc metastases in hTEBCs (5 lesions in total for each group) nor murine bone (6 and 4 lesions, respectively).

Furthermore, the differences in the size of metastases found in hTEBCs and murine bone indicate that PC3-Luc cells preferentially grew in the hTEBC compared with murine bone. Although the number of metastases was similar between the two bone compartments when the treatment was not effective (Denosumab and PBS group), the metastatic lesions found in the hTEBCs were on average twofold to fivefold larger than the ones in the murine bone. No differences in cell or colony morphology between metastatic lesions in murine femur and hTEBC were detected. PC3-Luc cells might have developed lesions in the hTEBC first, which allowed them to grow bigger in size. Representative histology (H&E) and IHC images from an animal of the PBS control group confirmed those results (Fig. 5b).

## Discussion

Ever since Steven Paget’s seed-and-soil hypothesis in 1889,^36^ species-specific interactions between cancer cells and their surroundings have proven to be of high importance for the growth of bone tumors as well as bone metastasis.^37^ As shown by us and others, bidirectional interactions between cancer cells and their extracellular matrix or with other cell types are crucial for tumor progression and metastatic spread in the osseous compartment.^8,28,38–40^ Generating a humanized microenvironment in several tissues or organs within one mouse model, e.g., prostate,^41^ immune system,^42^ or bone compartment,^43^ can narrow the biological gap between animal model and human patient. Eventually, this will allow us to use those models as a translatable drug testing platform.

In this study, we evaluated the translational potential of our humanized mouse model for PCa bone metastasis with the clinically approved treatments Denosumab and ZA. This model has already been used to study the species-specific interactions between human PCa cells and humanized niche for cancer bone metastasis.^28,41,44^

In our experimental PCa metastasis study, we observed a notable antitumor effect of ZA up to week 16 via whole body BLI. From week 17 onwards, animals treated with ZA, Denosumab or PBS showed a similar tumor burden by in vivo BLI. Denosumab treatment did not significantly decrease the tumor burden at any time point of the study, indicating that our model is not fully capable of recapitulating the effects of a human-specific treatment. However, as already described by Tannehill-Gregg et al.,^45^ in vivo BLI is not considered the most accurate method to determine the therapeutic effect of bone metastasis-targeting drugs. For such applications, the region of interest (humanized and murine bone) is covered by a considerable layer of different tissues (fur, skin, muscle, and fat), which can lead to inconclusive readouts.^46^ In order to make this method more powerful for the longitudinal monitoring of bone metastases especially in mouse bones, different planes and/or angles of imaging for each animal would have to be implemented in future studies.

Further analysis was conducted using ex vivo BLI, histology, and IHC, whereby the latter provides more detailed information on a cellular level within the tissue. Human PCa bone metastasis to both hTEBCs and murine femora were confirmed with ex vivo BLI and IHC. However, for hTEBCs the results of the two methods are much more comparable. The distribution of the individual values, as well as the statistical significance of both data sets, was similar for hTEBCs, but not for murine femora. In contrast to the murine femur, the hTEBC is located directly underneath the skin. Therefore, the hTEBC is covered by less tissue layers, which evades some of the drawbacks associated with in vivo BLI imaging of mouse bones. Thus, IHC confirmed the ex vivo BLI data of metastases to the hTEBCs, but not to mouse femora. In addition, the hTEBC allows access to the bone microenvironment and investigation of cancer bone metastases in real time enabling serial sampling.^47^

Both, ex vivo BLI and TRAP staining confirmed that ZA reduced the metastatic load significantly in hTEBCs, but not in mouse femora. This indicates that in our study the murine bone microenvironment has a substantially different molecular bearing on human PC3-Luc cells than the humanized microenvironment in the hTEBC. The mode of action of bisphosphonates is not species-specific,^48^ which suggests that the observed difference in the efficacy of ZA treatment is caused by the microenvironment. Also, Thudi et al.^49^ observed that ZA does not reduce the incidence of bone metastases in a nude mouse model of spontaneous metastasis using a canine PCa cell line. This group concluded that the effect of ZA on the reduction and inhibition of PCa metastasis depends on the biology of the respective cell line. Taking our results into account, the biology of the PCa cells and thus their behavior might rather depend on the microenvironment to which the primary tumor and/or distant bone metastases are exposed. This clearly highlights the importance of humanized models for preclinical research on PCa bone metastasis.

The values for total flux ex vivo and TRAP-positive tissue area were consistently higher in hTEBCs than in mouse femora. This points toward a higher number of human PCa cells invading the humanized bone microenvironment compared with murine bone. Area analysis of IHC staining confirmed that the hTEBC is preferred as a bone niche for metastatic growth by human PC3-Luc cells over the murine femur. Metastatic lesions in the hTEBC were found to be larger compared with the mouse femur at the endpoint of the study. This indicates that the PCa cells either migrated to the humanized bone microenvironment at an earlier time point, allowing them to reach a bigger size, or that the metastatic growth of human PCa cells is generally favored in the humanized bone microenvironment. Various inherent tissue characteristics of the hTEBC and the murine femur could be the underlying reasons for this interesting observation. Stiffness, vascularization, and species-specific molecular clues of the tissue can influence the initial outgrowth as well as the proliferation of PCa cells as first postulated in the seed-and-soil hypothesis.^36^ It is well-known for breast cancer that the stiffness of the breast tissue is a crucial factor when it comes to therapy resistance.^50^ Furthermore, Horas et al. showed that the loss of the vitamin D receptor promotes the skeletal colonization behavior as well as the expression of epithelial to mesenchymal transition (EMT) cell markers of breast cancer cells.^51^ We hypothesize that the hTEBC is more attractive for PCa cells due to the humanized microenevironment provided. In order to elucidate this hypothesis and to prove that these observations are caused by a temporal and not a spatial effect, additional in vivo studies with serial sampling at different time points are suggested.

Havens et al.^52^ demonstrated in a spontaneous metastasis SCID model with subcutaneous sponges mimicking a primary tumor, that the murine femur is the primary skeletal target of PC3 bone metastases. More than > 1.1 × 10^9^ human PCa cells were detected in the femur by human *Alu* qPCR.^52^ In the murine pelvis, ~0.9 × 10^9^ cells were found followed by the calvaria with > 0.7 × 10^9^ cells as the third most preferred murine skeletal site for human PCa cells.^52^ We could show that the humanized bone niche provided by the hTEBC is a more attractive soil for human PC3 cells than the murine femur. It has been well established in both in vivo^37^ and in vitro^53^ models that the host microenvironment determines the behavior of PCa cells via species-specific cel–cell interactions, adhesion molecules, proteases, and cytokines.^54^ Thus, it is essential to mimic the human bone microenvironment as closely as possible in any preclinical model used for the delineation of PCa bone metastasis. Paindelli et al.^55^ recently showed in a new 3D in vitro model for PCa bone metastasis how crucial it is to accurately recapitulate the core elements of the human bone stroma in order to mimic therapy responses. We and others focused on the generation of a metastatic niche for PCa bone metastases in vivo. Aguado et al.^56^ used so-called oncomaterials to engineer a pre-metastatic niche in mice, aiming to capture disseminated cancer cells. This work had its main emphasis on the involvement and effects of the immune system,^56^ which next to the bone microenvironment is another important compartment involved in the development of PCa bone metastasis. In this regard, we have shown previously that our model contains a morphological and biological functional bone organ with a metabolically active human bone microenvironment serving as a niche for PCa metastasis.^28^

Neither ex vivo BLI nor TRAP staining confirmed a therapeutic effect of Denosumab in humanized or murine bone in our model. The preclinical evaluation of this therapeutic Ab was challenging since it only binds to human RANKL, not the murine equivalent. Therefore, Kostenuik et al.^26^ developed a genetically modified mouse model expressing human RANKL. The establishment of a genetically engineered mouse model is costly and time consuming.^57^ Furthermore, in the case of the huRANKL mice, the genetic modification only provides a single human protein in a completely murine microenvironmental background and has significant effects on the murine physiology.^27^ Our hTEBC model is able to eliminate such inherent species difference problems without the need for the generation of complex genetically engineered mouse models when developing new treatment strategies for cancer bone metastasis.^57^ In the current stage, our mouse model provides a humanized bone microenvironment with multiple molecular factors relevant for human PCa metastasis, contrasting a single human ligand in the huRANKL mice. However, it does not account for the crucial role of a humanized immune system and a humanized prostate microenvironment. The humanized immune system,^32,47^ as well as the humanized prostate microenvironment,^41^ have been addressed by our group already in individual studies. The next step will be to combine the three humanized components to generate a next-generation model.

Here, we were able to demonstrate that human PCa cells grow preferentially in the hTEBC when compared with the murine femur. Furthermore, the hTEBC recapitulates the therapeutic effect of ZA more accurate than the murine femur. In the latter, no antimetastatic effect of the nonspecific anti-osteolytic bisphosphonate ZA was seen in our study. Altogether, these results provide a basis for future studies to develop next-generation animal models for the testing of treatments for PCa and other malignancies associated with bone metastases.

## Methods

### Preparation of the hTEBC

An inclusive protocol of the manufacturing and characterization of the hTEBC has been published by our group earlier.^32^ Briefly, mPCL scaffolds (5 mm diameter, 5 mm length) consisting of 600 fiber pairs were fabricated by melt-electrowriting,^58^ followed by calcium phosphate (CaP) coating.^59^ After informed consent, hOBs were obtained during hip arthroplasty in human donors (approved by the human ethics committee of the Queensland University of Technology (QUT)—#1400001024). Isolation and culture protocols have been described previously.^60^ The sterile mPCL-CaP scaffolds were seeded with 5 × 10^4^ primary hOBs. After 2 weeks, osteogenic culture conditions (α-MEM supplemented with 10 mmol·L^−1^ β-glycerophosphate, 50 μg·mL^−1^ ascorbic acid, 100 mmol·L^−1^ dexamethasone (all from Sigma-Aldrich), 100 IU·mL^−1^ penicillin and 100 µg·mL^−1^ streptomycin, and 10% FBS) were introduced to induce osteogenic differentiation and scaffolds were cultured until implantation.

The manufacturing of cell-laden GelMA hydrogels for in vitro and in vivo applications has been described previously.^61^ GelMA with a low degree of functionalization was dissolved in PBS and 1.5 mg·mL^−1^ lithium phenyl-2,4,6-trimethylbenzoylphosphinate (LAP) photo-initiator was added to obtain the precursor solution with a final concentration of 5% GelMA. HUVECs transduced with mCherry were cultured in ECGM-2 (from PromoCell and 100 IU·mL^−1^ penicillin and 100 µg·mL^−1^ streptomycin) and MSCs transduced with GFP and were cultured in DMEM with 16.5% FBS and 100 IU·mL^−1^ penicillin and 100 µg·mL^−1^ streptomycin). Both cell types were added to the precursor solution in a 10:1 ratio (6 × 10^6^ HUVECs per ml and 6 × 10^5^ MSCs per mL). Non-coated tubular mPCL scaffolds (3 mm diameter, 5 mm length, 250 layers) were placed upright in a custom-made Teflon cast. After casting the GelMA-cell solution into the scaffolds, the mold was covered with a glass slide. The gels were cross-linked for 40 s at 405 nm and washed in PBS. Finally, the gels were cultured until implantation in ECGM-2 containing the manufacturer provided supplement pack, 100 IU·mL^−1^ penicillin and 100 µg·mL^−1^ streptomycin and 125 ng·mL^−1^ SDF-1α, VEGF and FGF2 (all from Life Technologies) each.

### In vivo treatment study

The animal study was approved by the University of Queensland Animal Ethics Committee (QUT/591/16) and conducted in accordance with the Australian Code of Practice for the Care and Use of Animals for Scientific Purposes. Male NSG mice over 6 weeks of age were obtained from the internal breeding colony at TRI (led by A/Prof Pamela Pollock; TRI/376/12/WESLEY/CCQ/BREED). The animals were held under specific pathogen-free, temperature-controlled conditions and supplied with sterilized food and water at the Biological Resources facility at the Translational Research Institute (TRI; Brisbane, Australia). To subcutaneously (s.c.) implant the hTEBCs, two incisions were made longitudinally on the back of the animal and subcutaneous pockets in the required size were developed with blunt-ended scissors. The GelMA scaffolds were placed inside the human osteoblast scaffolds, the interspace was filled with 20 μL fibrin glue (TISSEEL Fibrin Sealant, Baxter Healthcare International) and 15 μL rhBMP-2 (1.5 µg·mL^−1^, Medtronic, Minneapolis, USA), hTEBCs were implanted and wounds were closed. After 12 weeks of in vivo bone tissue formation, all mice were intracardially injected with 1 × 10^6^ PC3-Luc cells (PC3 cells transduced with a lentivirus to express luciferase). The injection into the left ventricle was monitored with a VevoLAZR ultrasound system (FUJIFILM VisualSonics, Canada). Withdrawal of blood after the injection indicated successful delivery of the PCa cells into the blood stream. Distribution and metastatic outgrowth of the inoculated human PCa cells were monitored weekly via BLI. During week 13–18, animals received an intraperitoneal injection of 100 µg·kg^−1^ ZA (BioVision Inc., USA) or PBS or subcutaneous injection of 5 mg·kg^−1^ Denosumab (Xgeva; Amgen, USA) twice weekly. After 6 weeks of treatment, mice were euthanized by CO_2_ asphyxiation. The hTEBCs, mouse bones, and organs were explanted and ex vivo BLI imaging was performed. Samples were fixed in 4% PFA for 24 h at 4 °C and transferred into 80% ethanol for subsequent analysis.

### Monitoring of mineralized tissue formation

Mice were imaged on an UltraFocus 100 x-ray system (Faxitron, Tucson, USA) to follow bone formation at the hTEBC.

Also, mineralized tissue formation was monitored fortnightly by in vivo CT using the Inveon Micro-CT/PET Image Station (Siemens, Munich, Germany). The CT settings were as follows: 80 kV at 500 μA with a 0.5 mm aluminum filter, binning factor 2, 1 100 ms exposure time per projection, 180 projections at 360° rotation, and medium system magnification (source-to-center = 184.24 mm; source-to-detector = 345.34 mm). The standard Feldkamp algorithm for cone-beam CT reconstruction was used for image reconstruction. A Shepp–Logan filter as well as noise and ring artifact reduction were applied, resulting in an image matrix of 1 024 × 1 024 × 1 536 with an isotropic voxel size of 35.84 μm. Scans were analysed using ImageJ at a lower gray scale value threshold of 400.

Ex vivo CT analysis was performed to determine the mineralized tissue volume in all hTEBCs and to look for any metastatic lesions in the scaffolds and mouse bones. The samples were scanned with a Scanco Medical μCT 40 (Scanco, Switzerland) at a voxel size of 16 μm, intensity of 145 μA, and voltage of 55 kV. Reconstructions were assessed at a cut-off of 255 HU and volume and density of the formed mineralized tissue were quantified.

### Bioluminescence imaging (BLI)

To observe metastatic spread and outgrowth of luciferase transduced PC3-Luc cells, mice were imaged weekly with an IVIS Spectrum 200 (PerkinElmer, USA). Therefore, animals were intraperitoneally injected with 200 μL of a 7.5 mg·mL^−1^ solution of D-luciferin potassium salt (1.5 mg total) and anaesthetized (2% Isoflurane, 1 L·min^−1^ 100% O_2_). At the experimental endpoint ex vivo BLI of hTEBCs, the mouse skeleton and all inner organs were performed postmortem. All scans were assessed and quantified using LivingImage software (Version 4.5, PerkinElmer).

### Histology, IHC, and Tartrate-resistant acid phosphatase (TRAP) staining

After µCT analysis, mouse bones and hTEBCs were decalcified in a solution of 10% EDTA pH 7.4 at 37˚C. After automated dehydration in an Excelsior ES tissue processor (Thermo Fisher Scientific), specimens were embedded in paraffin. Tissue sections were used for (i) H&E staining, to evaluate general tissue structure and morphology, (ii) IHC analysis of the expression of human LaminAC (1:300, ab108595, Abcam), CD44 (1:75, H4C4, DSHB) and Ki67 (1:100, M7240, Dako), and (iii) staining of TRAP, a marker that is generally associated with osteoclast activity, but is meanwhile known to also play a role in the immune system.^62^

For each hTEBC construct, 20 sections were cut and for each mouse femur 40 sections. The 1st, 10th, and 20th section of each hTEBC (three slides total) and the 1st, 10th, 20th, 30th, and 40th section of each femur specimen (five slides total) were histologically analysed using H&E.

Paraffin sections were deparaffinized in xylene and rehydrated in an ethanol series for IHC. Antigen retrieval was conducted using heat mediated epitope retrieval with either sodium citrate (pH 6) or Tris-EDTA (pH 9) buffer. Endogenous peroxidase activity was blocked with 3% H_2_O_2_ (Sigma-Aldrich), Tissue sections were blocked with 2% BSA/PBS (Sigma-Aldrich) and subsequently incubated with the primary Ab for 1 h at RT or O/N at 4 °C. Signal detection and development were performed with the Envision + Dual Link secondary HRP system (Dako) and 3,3′-diaminobenzidine chromogen substrate (Dako) before counterstaining with Mayer’s haematoxylin (Sigma-Aldrich).

For TRAP staining, sections were deparaffinized and incubated with 0.1 mol·L^−1^ acetate buffer for 20 min at RT. Subsequently, samples were incubated with the staining solution, containing 0.5 mg·mL^−1^ naphtol AS-MS phosphate disodium salt, and 1.1 mg·mL^−1^ Fast Red TR salt (both from Sigma-Aldrich), for 1.5 h at 37 °C. Following Mayer’s haematoxylin counterstain, slides were manually cover slipped with aqueous mounting medium (ClearMount; ProSciTech, Australia).

Semi-automated quantification of IHC and TRAP staining was performed with Visiopharm 2017.2 software (Visiopharm, Denmark).

### Statistical analysis

Statistical analysis was conducted using IBM SPSS Statistics software Version 25 (IBM, New York, USA). The UNIANOVA model was used for the analysis of regression and variance for one dependent variable by one or more variables.^63^ To improve the robustness of the estimates of confidence intervals bootstrapping was included. The level of significance was set at *P* ≤ 0.05. For descriptive statistics, values were reported as the mean ± SEM or boxplots. Using GraphPad Prism (Version 7.00), the Tukey method was applied to all boxplots to determine the presence of values considered as outliers according to this method.^33^ If any outliers were detected, the respective data points were identified in the plot and excluded from further statistical analysis.
