# Correction: Humanized bone facilitates prostate cancer metastasis and recapitulates therapeutic effects of Zoledronic acid in vivo

**DOI:** 10.1038/s41413-020-0092-5

**Published:** 2020-04-03

**Authors:** Marietta Landgraf, Christoph A. Lahr, Alvaro Sanchez-Herrero, Christoph Meinert, Ali Shokoohmand, Pamela M. Pollock, Dietmar W. Hutmacher, Abbas Shafiee, Jacqui A. McGovern

**Affiliations:** 1 Centre in Regenerative Medicine, Institute of Health and Biomedical Innovation, Queensland University of Technology, Brisbane, Australia; 2 School of Biomedical Science, Institute of Health and Biomedical Innovation, Translational Research Institute, Queensland University of Technology, Brisbane, Australia; 3 Australian Research Council (ARC) Training Centre in Additive Biomanufacturing, Queensland University of Technology, Brisbane, Australia; 4 UQ Diamantina Institute, Translational Research Institute, The University of Queensland, Brisbane, QLD Australia

**Keywords:** Bone cancer, Bone, Bone cancer

**Correction to:** *Bone Research* 10.1038/s41413-019-0072-9, published online 21 October 2019

During a re-read of our article^1^ previously published in *Bone Research*, we regrettably found a mistake in the methods section relating to the concentration of recombinant human bone morphogenetic protein-2 (rhBMP-2) used to prepare the humanized tissue-engineered bone construct (hTEBC) for in vivo implantation. We used rhBMP-2 at a concentration of 1.5 μg·μL^−1^, rather than the originally stated rhBMP-2 concentration of 1.5 μg·mL^−1^. Although this correction does not affect the results or conclusions of the above paper, the authors agree to rectify this mistake by providing the correct methods paragraph below. We apologize for any inconvenience caused.

## Methods

### In vivo treatment study

The animal study was approved by the University of Queensland Animal Ethics Committee (QUT/591/16) and conducted in accordance with the Australian Code of Practice for the Care and Use of Animals for Scientific Purposes. Male NSG mice over 6 weeks of age were obtained from the internal breeding colony at TRI (led by A/Prof Pamela Pollock; TRI/376/12/WESLEY/CCQ/BREED). The animals were held under specific pathogen-free, temperature-controlled conditions and supplied with sterilized food and water at the Biological Resources facility at the Translational Research Institute (Brisbane, Australia). To subcutaneously (s.c.) implant the hTEBCs, two incisions were made longitudinally on the back of the animal and subcutaneous pockets in the required size were developed with blunt-ended scissors. The GelMA scaffolds were placed inside the human osteoblast scaffolds, the interspace was filled with 20 μL fibrin glue (TISSEEL Fibrin Sealant, Baxter Healthcare International) and 15 μL rhBMP-2 (1.5 μg·μL^−1^, Medtronic, Minneapolis, USA), hTEBCs were implanted and wounds were closed. After 12 weeks of in vivo bone tissue formation, all mice were intracardially injected with 1 × 10^6^ PC3-Luc cells (PC3 cells transduced with a lentivirus to express luciferase). The injection into the left ventricle was monitored with a VevoLAZR ultrasound system (FUJIFILM VisualSonics, Canada). Withdrawal of blood after the injection indicated successful delivery of the PCa cells into the blood stream. Distribution and metastatic outgrowth of the inoculated human PCa cells were monitored weekly via bioluminescence imaging (BLI). During week 13 to 18, animals received an intraperitoneal injection of 100 μg·kg^−1^ Zoledronic acid (ZA; BioVision Inc., USA) or PBS or subcutaneous injection of 5 mg·kg^−1^ Denosumab (Xgeva; Amgen, USA) twice weekly. After 6 weeks of treatment, mice were euthanized by CO_2_ asphyxiation. The hTEBCs, mouse bones and organs were explanted and ex vivo BLI imaging was performed. Samples were fixed in 4% PFA for 24 h at 4 °C and transferred into 80% ethanol for subsequent analysis.
